# Affective modulation of the associative-limbic subthalamic nucleus: deep brain stimulation in obsessive–compulsive disorder

**DOI:** 10.1038/s41398-019-0404-y

**Published:** 2019-02-04

**Authors:** Mircea Polosan, Fabien Droux, Astrid Kibleur, Stephan Chabardes, Thierry Bougerol, Olivier David, Paul Krack, Valerie Voon

**Affiliations:** 1 Univ. Grenoble Alpes, F-38000 Grenoble, France; 2 Inserm, U1216, Grenoble Institut des Neurosciences, F-38000 Grenoble, France; 3 Psychiatry Department, CHU Grenoble Alpes, F-38000 Grenoble, France; 4 Neurosurgery Department, CHU Grenoble Alpes, F-38000 Grenoble, France; 5 Division of Movement Disorder, Department of Neurology, Inselspital, University Hospital Bern, CH-3010, Bern, Switzerland; 6 Department of Psychiatry, University of Cambridge, Addenbrooke’s Hospital, Cambridge, CB2 0QQ UK; 7 Cambridgeshire and Peterborough NHS Foundation Trust, Cambridge, UK

## Abstract

Affective states underlie daily decision-making and pathological behaviours relevant to obsessive–compulsive disorders (OCD), mood disorders and addictions. Deep brain stimulation targeting the motor and associative-limbic subthalamic nucleus (STN) has been shown to be effective for Parkinson’s disease (PD) and OCD, respectively. Cognitive and electrophysiological studies in PD showed responses of the motor STN to emotional stimuli, impairments in recognition of negative affective states and modulation of the intensity of subjective emotion. Here we studied whether the stimulation of the associative-limbic STN in OCD influences the subjective emotion to low-intensity positive and negative images and how this relates to clinical symptoms. We assessed 10 OCD patients with on and off STN DBS in a double-blind randomized manner by recording ratings of valence and arousal to low- and high-intensity positive and negative emotional images. STN stimulation increased positive ratings and decreased negative ratings to low-intensity positive and negative stimuli, respectively, relative to off stimulation. We also show that the change in severity of obsessive–compulsive symptoms pre- versus post-operatively interacts with both DBS and valence ratings. We show that stimulation of the associative-limbic STN might influence the negative cognitive bias in OCD and decreasing the negative appraisal of emotional stimuli with a possible relationship with clinical outcomes. That the effect is specific to low intensity might suggest a role of uncertainty or conflict related to competing interpretations of image intensity. These findings may have implications for the therapeutic efficacy of DBS.

## Introduction

Affective states underlie daily decision-making and pathological behaviours relevant to obsessive–compulsive disorders (OCDs), mood disorders and addictions. Deep brain stimulation (DBS) is effective in Parkinson disease (PD)^1^ and OCD^2^ and critically offers the opportunity to investigate the neural underpinnings of cognitive and affective processes. DBS in OCD has shown efficacy in the anterior limb of the internal capsule (ALIC), the nucleus accumbens (NAcc) and ventral capsule/ventral striatum, and the subthalamic nucleus (STN)^2–4^. Effects on mood and anxiety are the most frequent stimulation-related reported side effects^5–7^. Thus, understanding the role of DBS on emotional processing may contribute to understanding the circuits underlying human emotional regulation and the mechanism of DBS in OCD.

Depressive reactions have been observed with acute stimulation in PD within different regions of the basal ganglia including the left^8^ or right substantia nigra^9^ and globus pallidus internus^10^. Acute induction of a positive emotional reaction (smile, laughter) intraoperatively during ALIC-NAcc DBS in OCD was suggested to predict DBS outcome^11^.

The STN is a small nucleus within the indirect pathway and receives significant hyper-direct prefrontal cortex connections highlighting its role as a nexus for integration^12,13^. The posterior dorsal motor STN is an effective target for PD and the anterior ventral limbic-associative STN^1^ is effective for OCD^2^. DBS targeting the motor STN in PD have reported acute positive emotion such as laughter/hilarity^14^ or euphoric manic behaviour^13,15^ and acute depressive reactions^16,17^, anger^18^, pathological crying^19^ and pseudo-bulbar crying^20^. These presumed acute stimulation effects of the STN have been suggested to be related to disinhibition of behaviour^5^ and may also be dependent upon baseline diagnoses^21^.

With chronic stimulation, STN DBS may be associated with frequent major depressive episodes (for review, see^5^) or apathy^22^, but reduction in dopaminergic pharmacotherapy is an important confounder. However, in contrast to discrete major depressive episodes, STN DBS in PD consistently improves overall depression^23^ and anxiety scores^5^. Post-operative hypomania/mania is the most consistently reported post-operative psychiatric stimulation-induced effect reported in PD (4%)^6^, which may be linked to antero-ventral STN stimulation^13^ and can be clinically addressed by decreasing voltage, dopaminergic dose or changing stimulation contacts dorsally^24^. Similarly, in OCD, mood effects of antero-ventral STN DBS have involved hypomania rather than depressive symptoms^2^. Stimulation duration may also be relevant: acute effects may involve euphoric feelings and improved motivation, which are less likely with chronic STN stimulation in PD^16,22,25,26^. Thus, the short-term^2^ and suggested long-term^27^ clinical benefit of antero-ventral STN DBS in OCD may not be dependent on mood impact although may influence quality of life^28^.

OCD is characterized by obsessions or repetitive intrusive thoughts and urges leading to compulsions or behaviours, which subjects feel driven to perform. Impairments in the processing of emotional stimuli in OCD with a more negative (or less positive) appraisal of emotional stimuli have been reported^29,30^, suggested to be related to a generalized negative appraisal bias. Studies on STN stimulation-locked emotional processing have focused on the motor STN in PD (for a review, see^5^); how DBS alters limbic function in OCD patients targeting the associative-limbic STN remains an open question. Here we sought to assess the role of the antero-ventral associative-limbic STN in limbic processing. We assessed the intensity of valence and arousal ratings in both pleasant and unpleasant imagery and divided the International Affective Picture System (IAPS)^31^ images into low and high valence intensity presuming that low valence images may have less ceiling effect and hence more sensitive to subjective interpretation and capacity for change. We hypothesized that STN DBS would increase subjective pleasant and decrease subjective unpleasant scores particularly with lower valence images.

## Methods

### Participants

Twelve OCD subjects were recruited from Grenoble University Hospital, tested On and Off DBS and compared with 24 healthy volunteers (HVs). OCD patients (eight females; mean age: 41.75 ± 7.94 years) had undergone bilateral STN DBS for mean 38.1 ± 18.8 months prior to testing (duration of the stimulation range prior to the study: 5–71 months). Patient characteristics are shown in Table 1. Disease duration before surgery was 18 ± 9.2 years, and Yale Brown Obsessive–Compulsive Scale (YBOCS) score (assessing OCD severity^32^) before surgery was 34.3 ± 3.2. At the time of the study, YBOCS baseline score was 20 ± 9.1 with a clinical improvement (compared with pre-surgery state) of 41 ± 28%. Patients had at least 5 years of treatment-resistant, severe, disabling OCD before DBS surgery. Several patients had some neuropsychiatric comorbidities (obsessive–compulsive spectra): one subject have comorbid Tourette’s syndrome; another subject have comorbid skin picking and another subject had a premorbid history of an eating disorder that was in remission 20 years before surgery. One patient had hypersomnia. All subjects were right handed and had normal or corrected to normal vision.

**Table 1:** Clinical and demographical characteristics of the OCD patients

Patient number/age (years)/gender (F/M)	Age at surgery (years)	Duration of disease before surgery (years)	Age at onset of OCD (years)	Duration of DBS (months)	YBOCS before surgery	YBOCS Baseline at time of study	Medications at the time of the study
1/46/M	39	18	21	71	37	25	Fluvoxamine 200 mg/day; Lorazepam 4 mg/day
2/49/F	42	25	17	64	30	28	Aripiprazole 30 mg/day; Olanzapine 5 mg/day; Escitalopram 20 mg/day; Clomipramine 75 mg/day
3/39/M	36	17	19	32	32	28	Paroxetine 60 mg/day
4/53/F	49	39	10	51	35	29	Fluoxetine 20 mg/day; Clomipramine 25 mg/day
5/37/M	34	13	21	22	32	27	Clomipramine 150 mg/day; Oxazepam 175 mg/day; Alimemazine 50 mg/day
6/41/F	38	11	27	35	36	6	None
7/43/F	40	15	25	32	36	23	Fluvoxamine 200 mg/day; Hydroxyzine 50 mg/day; Clomipramine 25 mg/day
8/41/F	37	5	32	44	32	2	Venlafaxine 37.5 mg/day; Clotiazepam1.5 mg/day
9/30/M	27	10	17	25	38	24	Sertraline 50 mg/day; Aripiprazole 20 mg/day; Methylphenidate 60 mg/day; Pitolisant 20 mg/day
10/56/F	52	25	27	51	40	18	Zopiclone 7.5 mg/day; Aripiprazole 2.5 mg/day; Hydroxyzine 100 mg/day
11/33/F	33	21	12	5	30	11	Venlafaxine 150 mg/day
12/33/F	33	26	7	25	34	19	Fluoxetine 20 mg/day; Levothyroxine 125 µg/day

*M* male, *F* female, *YBOCS* Yale Brown Obsessive–Compulsive Scale, *DBS* deep brain stimulation, *OCD* obsessive–compulsive disorder

The OCD subjects were implanted bilaterally with two electrodes 3389 connected to a Kinetra stimulator (Medtronic, Minneapolis, Minnesota, USA), accordingly to the STN DBS protocol already published elsewhere^2,4^. The surgical procedure targeted the antero-ventral non-motor part of the STN. Indirect targeting was defined as 1 mm anterior to the mid-commissural point, 10 mm lateral from the midline and 4 mm below the AC-PC line. The final target was adapted laterally according to the visualization of the medial border of the STN. The antero–posterior coordinates were defined 2 mm anterior to the anterior border of the red nucleus. Stimulation frequency and pulse width were set at 130 Hz and 60 µs, respectively; stimulation voltage and activated contacts were adjusted individually to obtain the best clinical response.

The 24 age-matched (± 5y) and gender-matched HVs (16 females; 42.67 ± 8.34 years old) were recruited from the University and community in Grenoble. Subjects were screened by a psychiatrist with the Structured Clinical Interview (SCID) for DSM IV in order to check the exclusion criteria. Exclusion criteria for HVs were past or present serious psychiatric or medical disorders, as well as any psychotropic medications. The research protocol was approved by the Ethics Committee of Grenoble University Hospital (ancillary study to protocol N° ID RCB: 2012-A00490-43). All participants volunteered to participate in the study and gave written informed consent.

### Procedure

DBS has the advantage of reversibility, which enables studying its effects within DBS On/Off paradigms. Thus, the patients performed the tasks with STN-DBS On and with STN-DBS Off, in a randomized double-blind within-subject design over 2 successive days to allow a sufficiently long washout of DBS effects (Table 2). All patients were under STN DBS On when included in the study. Patients were randomized (stratification by gender) to one of two arms: (1) DBS was switched off in the morning of Day 1 and patient tested 4 h later; DBS was kept off overnight and in the morning of Day 2 the DBS was switched on and the patient was tested again 4 h later (after 4 h DBS on). (2) Alternatively, in the other arm, DBS was kept On on Day 1 and patient tested similarly at the same moment of the day, 4 h after the blind control of his DBS device; the DBS was kept on overnight and, in the morning of Day 2, the DBS was switched off and patient tested 4 h later. Both the tester and patient were blinded to the condition of stimulation (the control of the DBS device and the DBS switches were performed by another investigator). The blind was maintained during the study as patients were unable to identify properly the stimulation condition (On/Off). The patients continued their usual medication during the study. HVs were tested once.

**Table 2:** Study design for OCD subjects

Day 1	Day 2
9 a.m.: YBOCS (baseline)	9 a.m.: YBOCS
10 a.m.: DBS device control	10 a.m.: DBS device control
A. On	A. Switch Off
B. Switch Off	B. Switch On
2 p.m.: IAPS testing	2 p.m.: IAPS testing

*YBOCS* Yale Brown Obsessive–Compulsive Scale, *DBS* deep brain stimulation, *OCD* obsessive–compulsive disorder, *IAPS* International Affective Picture System

### Tasks

#### Affect task

Subjects were shown 20 visual stimuli out of a dataset from the International Affective Picture System (IAPS^31^). Subjects were shown neutral (four images) and high pleasant and unpleasant images (four images of each condition) and low pleasant and unpleasant images (four images of each condition). All the images were balanced between emotional categories (human, animals, objects, scenery) and were presented on a computer screen in a pseudorandomized order with no time limitation for responding. The image ceased when the subject responded with an interstimulus interval of 2 s during which a fixation-cross was presented in the screen’s centre. Each image was presented once. Subjects were instructed to look at the picture and rate the degree of pleasantness and unpleasantness (emotional valence) by moving the mouse cursor along a line anchored at 0 (unpleasant emotion) to 5 (neutral) and 10 (pleasant emotion) (visual analogue scale). A similar procedure was conducted for degree of arousal. The arousal referred to the intensity of the emotional activation: from calm (score 0) to very excited (score 10). We focused on the outcome measure of ratings of pleasantness and arousal.

### Statistical analysis

The behavioural scores were first assessed for outliers and normality of distribution using Shapiro–Wilkes test. We first compared the effects of On and Off DBS on the neutral stimuli to assess the influence of stimulation on neutral images using a paired *t-*test prior to focusing on the affective stimuli. We then compared the scores for affective ratings (emotional valence) and arousal ratings separately using a repeated-measures analysis of variance (ANOVA with a within-subject On-Off factor and within-subject Affect factor (Pleasant and Unpleasant). This was separately conducted for the high valence and low valence stimuli. The *p* < 0.05 was considered significant for the main hypothesis of a DBS effect focusing on low valence stimuli (Bonferroni correction for multiple corrections *p* < 0.0125). We then used a repeated-measures ANOVA to compare OCD patients On and Off DBS and HV with a within-subject Affect factor (Pleasant and Unpleasant) separately conducted for high and low valence stimuli. We further asked if the effect on valence was related to clinical parameters by including YBOCS change (pre–post) as a covariate in the repeated-measures ANOVA.

## Results

**Fig. 1:**
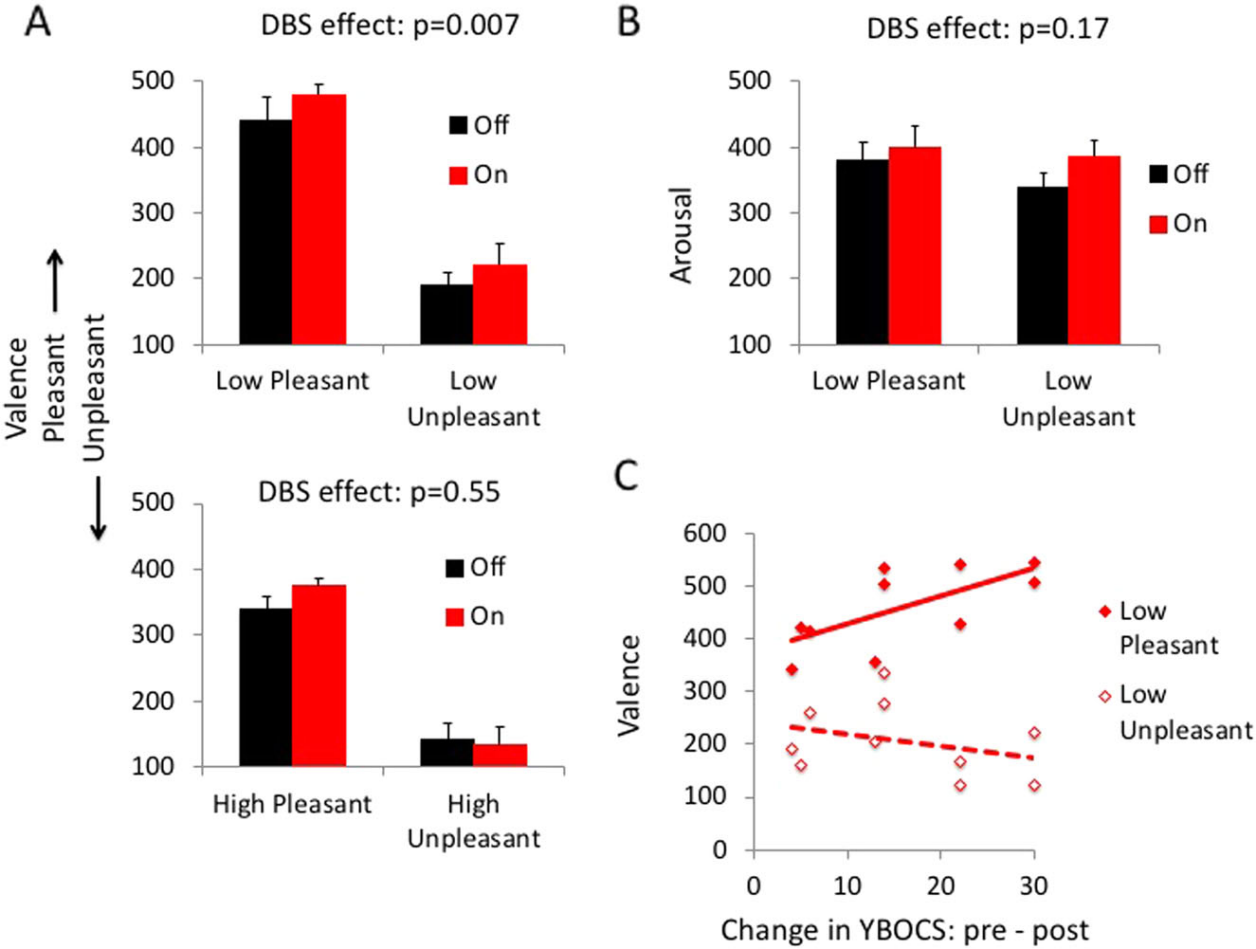
Subthalamic deep brain stimulation effects on affective valence and relationship to clinical outcomes. **a** Valence ratings for emotional images in obsessive-compulsive disorder (OCD) subjects On and Off deep brain stimulation (DBS) targeting the subthalamic nucleus. Top: low intensity. Bottom: high intensity. **b** Arousal ratings for low pleasant and low unpleasant emotional images. **c** Valence ratings for low pleasant and unpleasant images Off DBS as a function of change in pre-operative and post-operative Yale Brown Obsessive–Compulsive Scale (YBOCS). Error bars represent standard error of the mean

As two subjects did not complete the low valence component of the task due to an examiner error during testing, we restricted our analyses to the 10 OCD subjects who had completed the full task. The data were normally distributed. There were no effects of DBS on neutral stimuli (valence: *t* = –0.84, df = 9, *p* = 0.43; arousal: *t* = 0.14, df = 9, *p* = 0.89). In the critical within-subjects comparison of low valence stimuli, there was a main effect of valence (*F*(1, 9) = 63.11, *p* < 0.0001) and of DBS (*F*(1, 9) = 11.84, *p* = 0.007) but no interaction effect (*F*(1, 9) = 0.04, *p* = 0.85) (Fig. 1). OCD subjects On DBS had more positive ratings across both positive and negative valences relative to Off DBS. Although we had a hypothesis specific to the low valence stimuli, the findings were also significant after Bonferroni correction for multiple comparisons. This effect of STN DBS was specific to the low valence condition as in the comparison of high valence stimuli, there was a main effect of valence (*F*(1, 9) = 49.16, *p* < 0.0001) but no effect of DBS (*F*(1, 9) = 0.39, *p* = 0.55) or DBS × valence interaction (*F*(1, 9) = 2.79, *p* = 0.13) (Fig. 1).

In the assessment of arousal in the low valence condition, there was no effect of arousal (*F*(1, 9) = 0.69, *p* = 0.43), DBS (*F*(1, 9) = 2.26, *p* = 0.17) or DBS × arousal interaction (*F*(1, 9) = 0.71, *p* = 0.42) (Fig. 1). In the high valence condition, there was an effect of arousal (*F*(1, 9) = 5.96, *p* = 0.04), and no effect of DBS (*F*(1, 9) = 0.04, *p* = 0.84) or DBS × arousal interaction (*F*(1, 9) = 0.25, *p* = 0.63). We conducted a further assessment demonstrating using paired *t*-tests that there were no differences in subjective arousal between positive and negative stimuli Off stimulation (positive: 400.88 (80.95); negative: 431.47 (50.38), *t* = –1.30, df = 9, *p* = 0.23) as expected but the difference in arousal arose On stimulation (positive: 388.77 (60.20); negative: 433.42 (70.41), *t* = –2.49, df = 9, *p* = 0.03). We confirm that the subjective assessment of arousal in this patient group Off stimulation was no different as a function of valence consistent with IAPS arousal ratings. The effects On stimulation suggest a potential effect of stimulation on decreasing arousal of positive images; however, the DBS effects and interaction effects were both not significant.

To assess the effects of order of stimulation in the low valence condition, we conducted a separate analysis comparing low valence with Order (On-Off *N* = 5; Off-On *N* = 5) as a between-subjects factor. There were no effects of Order (*F*(1, 8) = 4.41, *p* = 0.07) and the DBS effects remained significant (*F*(1, 8) = 10.94, *p* = 0.01).

We then asked if there was a relationship between the low valence condition and change in YBOCS scores pre- and post-STN DBS by including the change in YBOCS scores (YBOCS change = pre–post) as a covariate. There was a main effect of DBS (*F*(1, 8) = 23.31, *p* = 0.001) and of valence (*F*(1, 8) = 8.07, *p* = 0.02) and no DBS × valence interaction (*F*(1, 8) = 0.54, *p* = 0.48). There was also an interaction between DBS × YBOCS change (*F*(1, 8) = 8.40, *p* = 0.02) and a valence × YBOCS change interaction (*F*(1, 8) = 9.02, *p* = 0.02). Given these interactions with YBOCS change, we conducted exploratory Pearson correlation analyses with YBOCS change. The change in YBOCS with stimulation (YBOCS at the time of experiment – YBOCS pre-op), the more pleasant the subjective ratings of the low pleasant stimuli Off DBS (Pearson correlation coefficient *R*^2^ = 0.67, *p* = 0.03) (Fig. 1) with no significant correlation observed On DBS or with low unpleasant (*p* > 0.05).

The repeated-measures ANOVAs comparing valence or arousal for all groups including healthy controls were not significant (*p* > 0.05).

We further assessed any relationship between the difference between On and Off in the current findings with risk taking choices to reward and loss, *K*-values of delay discounting and reflection impulsivity measures previously reported in this same population. As the risk choices were not normally distributed, Spearman rank correlation was used. No significant findings were noted.

## Discussion

We showed that STN DBS enhances positive (or decreases negative) subjective ratings of low-intensity stimuli irrespective of valence. This effect was not observed with high valence or arousal ratings. These findings may be related to greater variability and capacity for change in low valence ratings (rated subjectively more negative in the Off state with greater positive shift in the On state) with the high valence conditions demonstrating ceiling effects. We also show that the change in YBOCS or severity of OCD pre- versus post-op interacts with both DBS and valence ratings. On exploratory analysis, the greater the YBOCS improvement, the higher the valence ratings Off DBS in the low pleasant condition with no correlation observed On DBS. This is the first study to suggest a role for emotional processing of the antero-ventral STN with DBS in OCD patients with previous studies focusing on the STN in PD patients. These findings are consistent with our previous confirmation that the clinically optimal coordinates in this same OCD population converge within the antero-ventral associative-limbic STN with resting state functional connectivity with limbic and associative prefrontal regions^33,34^.

OCD is associated with impaired processing of emotional stimuli with a more negative (or less positive) appraisal of emotional stimuli. In response to both unpleasant and pleasant odours, OCD subjects showed both enhanced disgust sensitivity and intensity and greater insular activity correlating with severity and anxiety^35^. A perception bias towards negative facial emotion (disgust) was also observed in OCD^36^. Similarly, OCD subjects overestimate the valence of negative/unpleasant stimuli^30^, whereas pleasant stimuli were rated as less pleasant/more unpleasant. Along these lines, enhanced functional connectivity of a salience network was observed during early conditioning to a fearful threatening stimulus^37^ with impairments in ventromedial prefrontal cortex activity to conditioning to the safety signal. Together, these observations in OCD suggest that this negative cognitive bias underlying emotional appraisal and processing may be a generalized impairment that might underlie impairments in associative learning processes to negative, threatening or fearful stimuli^35^. Thus, targeting this negative cognitive bias and decreasing the negative (or less positive) appraisal of emotional stimuli irrespective of the valence may have therapeutic efficacy. Here we showed that stimulation of the anterior limbic-associative STN in OCD enhances the positive appraisal of low-intensity stimuli irrespective of valence, consistent with an improvement in negative cognitive bias.

That the effects were shown in the low valence stimuli, which may have greater range for subjective interpretation of intensity and capacity for change rather than the high valence stimuli with likely greater ceiling effects, may also implicate a role for uncertainty. Here uncertainty is defined as greater variance or alternative outcomes in subjective interpretation of intensity. Uncertainty has also been suggested to be relevant in OCD: greater accumulation of evidence has been reported to greater perceptual and probabilistic uncertainty in OCD^38–40^, although these findings are not always consistent^41^ or only significant after controlling for neuroticism^42^. Uncertainty (e.g., the possibility of alternative outcomes) has been suggested to increase the gathering excessive evidence to support their decision with some^43,44^ but not all studies^38,45^. Using a delayed matching-to-sample task with choice verification, poor insight triggered checking behaviours in OCD patients, which indexed uncertainty^46,47^. OCD subjects have also shown greater explicit subjective ratings of uncertainty for low but not higher uncertainty evidence in a probabilistic reasoning task^44^.

### Emotion processing and STN

Many but not all studies in PD patients have shown an effect of DBS targeting the motor STN on emotion recognition, particularly with negative emotions. In PD patients, tested 3 months before and after STN DBS, recognition of facial emotions was impaired^48,49^, particularly with fearful^50^ and angry faces^51^ and also with emotional prosody^48^. Disgust recognition was impaired on STN DBS and off Levodopa, whereas fear recognition was impaired in the off state in both therapies^52^. Combined DBS and Levodopa also improved general emotion recognition^53^. However, some studies reported no differences on emotion recognition as a function of DBS in both late^54^ and early PD^55^. The decoding of prosody was also impaired in patients irrespective of stimulator status; however, STN DBS was associated with more rapid responses to highly conflicting contradictory emotional stimuli suggested to reflect impaired inhibition of irrelevant stimulus dimensions with competing response alternatives^56^. This latter observation is consistent with the common observation of hastened responding and a decrease in the decision-making threshold with conflict with STN DBS^33,57,58^.

STN DBS also influences subjective intensity of valence mostly by decreasing the intensity of negative and enhancing positive stimuli. STN DBS in PD have been reported to enhance emotional intensity of prosody^48^, lower intensity to aversive stimuli^59,60^ and with ventral STN stimulation, enhance ratings of positive stimuli^61^. Our findings suggest a generalized effect irrespective of valence focusing particularly on low-intensity stimuli. We suggest that these may be more ecologically valid as much emotional stimuli in the environment are of low intensity and perhaps more amenable to shifts in subjective perception.

PD patients showed decreased alpha event-related desynchronization (ERD) to pleasant and unpleasant stimuli 1–2 s after stimuli onset^62,63^ with ERD correlating with individual subjective ratings. Alpha band (8–12 Hz) activity has been suggested to represent a marker of limbic activity in non-STN regions including the bed nucleus of stria terminalis or subgenual cingulate area, or pathologies such as major depressive disorder and OCD^64^. Beta oscillations have also been used to map dorsal oscillatory and ventral non-oscillatory STN activity^12^. Emotive auditory stimuli evoked ventral right STN activity suggesting both regional specialization and hemispheric asymmetry^65^. Similarly, enhanced activity to both angry and happy auditory stimuli was observed in the right STN with differential timing of activity^66^. Using perioperative micro-recordings, 17% of single STN neurons responded in the alpha band activity (500–2000 ms) to emotional stimuli^67^, a measure analogous to alpha band local field potential activity^62,63^. Affective neurons were recorded in the sensorimotor regions consistent with integration of functional STN territories with no evidence of laterality, but with a posterior valence and anterior arousal segregation^67^.

Neuroimaging studies in STN DBS PD patients have identified a neural network implicated in emotional processing. Decreased fear recognition correlated with decreased glucose metabolism of the right orbitofrontal cortex^68^. STN DBS increases anterior cingulate activity and decreases putaminal activity to emotional imagery^69^. DBS diminished frontal polar oxy-haemoglobin to positive stimuli and diminished frontopolar and right lateral prefrontal cortex and increased bilateral inferior ventrolateral prefrontal cortex oxy-haemoglobin to negative stimuli^70^. Finally, a fluorodeoxyglucose-positron emission tomography (FDG-PET) study showed that the decrease in intensity to disgust ratings after STN DBS in PD correlated with prefrontal–insular–cerebellar activity^71^.

## Limitations

This study is not without limitations. The sample size is low; however, we note that this population is very difficult to recruit and further, the randomized controlled trial study of STN DBS in OCD consisted of only 16 subjects. Notably, the within-subject design of this current study reduces inter-individual variability and tends to minimize the heterogeneity potentially related to the different types of OCD. Medication status can also interfere with emotional state, but this has not been modified during the study. Healthy controls were only tested once, which limits comparisons due to an order effect in the experimental condition.

## Conclusions

We suggest two plausible main mechanisms that might underlie these and our previous observations. We have previously shown that STN DBS enhances reflection impulsivity and delay discounting and decreases risk taking to rewards in the same population, an effect we suggested may be related to enhanced evidence accumulation in the context of conflict^33,34^. We did not show any relationship in our current findings with our previous findings on decisional impulsivity or risk taking^33,34^. However, since we observed a change only in low valence stimuli, our findings may similarly be related to a specific effect of STN DBS on greater competing interpretation alternatives and hence greater conflict^56^. That the direction of effect is an enhancement in positive intensity is in keeping with previous reports of STN DBS impairing negative emotion recognition and enhancing positive or decreasing negative intensity ratings. We have also previously reported dissociable findings on risk taking to rewards and losses with STN DBS influencing decreased risk taking to rewards and impairing discrimination of loss magnitude. In the latter, we suggested that impaired discrimination of loss magnitude may be specifically related to STN DBS influencing the indirect NoGo pathway mediated via low affinity D2 receptors^34^. Thus, STN DBS may impair subjective discrimination of negative value and recognition of negative affect and here we show a specific shift towards more subjective positive valence attribution away from negative. Whether the impairment may be specific to negative valence and value, or have an additional separate effect on positive valence is not clear. In either case, this will shift the cost–benefit analysis.

In conclusion, our findings support the ‘tuner' role of STN in emotional processing by decreasing the negative appraisal of low valence stimuli in OCD. These findings highlight potential mechanisms involved in the therapeutic benefit of STN DBS in OCD and suggesting its putative application in resistant major depression disorder.
